# A comparative assessment of plaque removal and toothbrushing compliance between a manual and an interactive power toothbrush among adolescents: a single-center, single-blind randomized controlled trial

**DOI:** 10.1186/s12903-018-0588-1

**Published:** 2018-08-03

**Authors:** Christina Erbe, Violetta Klees, Priscila Ferrari-Peron, Renzo A. Ccahuana-Vasquez, Hans Timm, Julie Grender, Pamela Cunningham, Ralf Adam, Svetlana Farrell, Heinrich Wehrbein

**Affiliations:** 1 Department of Orthodontics and Dentofacial Orthopedics, University Medical Center of the Johannes Gutenberg University, Augustusplatz 2, 55131 Mainz, Germany; 2 Oral Care Department, Procter & Gamble Company, Kronberg, Germany; 3 Global Oral Care Department, Procter & Gamble Company, Mason, OH USA

**Keywords:** Compliance, Toothbrushing behavior, Dental plaque, Technology, Oral hygiene, Interactive power toothbrush

## Abstract

**Background:**

Many adolescents have poor plaque control and sub-optimal toothbrushing behavior. Therefore, we compared the efficacy of an interactive power toothbrush (IPT) to a manual toothbrush (MT) for reducing dental plaque and improving toothbrushing compliance.

**Methods:**

In this randomized, parallel single-blind clinical study, adolescents brushed twice daily with either a MT (Oral-B® Indicator soft manual toothbrush) or an IPT (Oral-B® ProfessionalCare 6000 with Bluetooth). Subjects brushed for 2 min, plus an additional 10 s for each ‘Focus Care Area’. At screening and Week 2, afternoon pre-brushing plaque was assessed via the Turesky Modification of the Quigley-Hein Plaque Index (TMQHPI), and supervised brushing duration was measured.

**Results:**

Sixty subjects were randomized; 98% completed. At Week 2, the mean reduction in whole mouth plaque relative to baseline was 34% (*p* < 0.001) for the IPT versus 1.7% (*p* = 0.231) for the MT. For Focus Care Areas, the IPT yielded a 38.1% mean TMQHPI reduction (*p* < 0.001) versus 6.2% for the MT (*p* < 0.001). Mean brushing time versus baseline increased 34 s in the IPT group (*p* < 0.001) while remaining flat in the MT group (*p* = 1.0).

**Conclusions:**

Over 2 weeks, adolescents using an IPT experienced superior plaque reduction and increased overall brushing time versus those using a MT.

**Trial registration:**

This trial was retrospectively registered (ISRCTN10112852) on the 18th, June 2018.

## Background

The adolescent years are a time of significant change, from marked physical growth, to the assumption of greater personal responsibility. Self-care tasks formerly closely supervised by parents - like daily oral hygiene - are generally transitioned to the adolescent to manage as autonomy grows. Teens typically want to make a good impression with their peer group and are appearance-conscious, with the desire for an attractive smile [1]. Yet research relying on both self-report and videotaped observation has shown that many adolescents are not following the dental profession general consensus of a twice daily toothbrushing recommendation or the suggested two-minute brushing duration per session [2–4]. A recent Gallup Youth Survey revealed that 30 and 42% of surveyed teen girls and boys, respectively, reported brushing only once each day, and 2% of the group said they don’t even brush daily [5]. Previous studies have shown even poorer rates of compliance, with the lowest toothbrushing frequency in adolescent males [6, 7].

Compounding the challenge faced by adolescents in maintaining a healthy dentition and gingiva is the fact that they often show a predilection for carbohydrate-laden snacks, and sugary sodas or popular energy drinks [8–10], which could contribute to higher levels of dental plaque formation. Fixed orthodontic treatment is a rite of passage for many teens, complicating effective plaque removal [11]. If in addition, motivation is low and teen efforts at oral hygiene are substandard, the adolescent population becomes at high risk for caries and gingivitis. The 2011–2012 United States National Health And Nutrition Examination Survey found that by age 19, 67% of US teens have experienced tooth decay in the permanent dentition, with untreated decay in 20% [12]. A study of 889 Brazilian teens aged 15 to 19 years found a caries prevalence of 87.5% and gingivitis in 94.7% of the participants [13]. Similar higher than desirable caries and gingivitis prevalence in the adolescent demographic have been reported in varied geographies [14–16].

If thorough oral hygiene is not always a priority, one area where adolescents *do* focus substantial daily time and energy is on technology-based communication, including instant access to information via the web and peer interaction through social media. Life without these technologies has become largely unimaginable for most teens, with surveys like the Pew Research Center’s 2013 Teens and Technology reporting that 78% of American teens own a cell phone, roughly one-half of which are smartphones [17]. With the popularity of medical/health mobile applications (apps) for wireless devices at an all-time high comes the opportunity to harness the pervasive adolescent technology adoption to foster better oral health. Accordingly, Oral-B [Procter & Gamble, Cincinnati, OH, United States] has combined state-of-the-art wireless technology with clinically proven power toothbrushes in an innovative mobile platform to boost cleaning performance, increase patient motivation, and create better daily oral hygiene habits through real-time instant feedback and tracking. The interactive Oral-B® Professional Care 6000® with Bluetooth® 4.0 connectivity allows for two-way communication between the toothbrush and the smartphone-connected mobile app to enable instant feedback around brushing force and session length.

Power toothbrushes - once a novelty - are now mainstream and popular, and certain power brushes have repeatedly demonstrated plaque removal superiority compared to manual brushes in both laboratory and clinical investigations [18]. The Oral-B Professional Care 6000 employs the oscillating-rotating mode of action. A unique feature of this interactive brush is the ability for pre-programming of a personalized/customized app with the user’s specific dentition regions where clinical examination has revealed that more brushing attention may be needed (as shown by more excessive plaque build-up and/or gingivitis), termed ‘Focus Care Areas’. During a brushing session, the app reminds and guides the brusher to spend some additional time and effort on these areas of concern.

The central aim of this clinical investigation in adolescents was to assess whether brushing with an interactive power toothbrush would provide additional plaque removal efficacy beyond that achieved with a standard manual toothbrush, for both the whole dentition, and in individual subject Focus Care Areas. Additionally, this study sought to determine if the duration of toothbrushing as a part of brushing compliance would differ between adolescents who used an interactive power toothbrush, and those who used a standard manual toothbrush.

## Methods

### Study design and participants

This parallel group, randomized, controlled, two-week, single-blind clinical trial evaluated the relative plaque removal efficacy, as well as the duration, of toothbrushing with an interactive power toothbrush (equipped with Bluetooth technology and a smartphone application) compared to brushing with a manual toothbrush in adolescent volunteers. This study was performed at the Department of Orthodontics and Dentofacial Orthopaedics of the University Medical Center of the Johannes Gutenberg-University in Mainz, Germany between February and March 2015. The teenage participants who were enrolled were required to be between 13 and 17 years of age and in good general health, were pre-study manual toothbrush users, and showed evidence of plaque formation at the screening visit, with a specified minimum whole mouth mean plaque score. Qualified subjects also needed at least 16 natural teeth (excluding third molars), and at least one - but not more than four - ‘Focus Care Areas’ in the dentition, as identified by the study investigator, and defined below in **Clinical Evaluations**. The conditions which excluded volunteers from study enrollment included unfamiliarity with or inability to use smartphone technology, multiple and/or untreated caries, severe gingivitis or periodontitis requiring treatment, tobacco use, fixed orthodontic appliances, peri/oral piercings, antibiotic/chlorhexidine usage within 2 weeks of the screening visit and/or a dental prophylaxis within 4 weeks of the screening visit. Continuing subject eligibility based on the study entrance criteria was assessed at the onset of both post-screening study visits.

The Ethic-Commission of the State Medical Association Rhineland-Palatinate, Mainz, Germany [code 837.451.14 (9690)] reviewed and approved the study protocol and subject consent form in advance of the trial, in accordance with the ethical standards laid down in the 1964 Declaration of Helsinki and its later amendments. Each subject and his/her guardians provided written and informed consent prior to participation.

Subjects were alerted to restrictions on their pre-appointment activities, which applied to the screening and both subsequent study visits. They were cautioned to refrain from brushing their teeth and from performing any other oral hygiene procedures following their morning brushing (and no later than 8:00 AM) prior to each visit. Additionally, participants were directed to cease eating, drinking, or chewing gum for 2 h prior to their appointment time, other than small sips of water up to 45 min before the visit.

At the screening visit, subjects presented with their existing manual toothbrush. Their medical history was reviewed to confirm study eligibility according to the inclusion and exclusion criteria, and they received a comprehensive visual examination of the oral cavity and perioral area, including hard and soft tissues. Subjects then swished with disclosing solution (Mira-2-Ton®; Hager Werken, Germany) for 1 min. The clinical examiner evaluated the disclosed plaque to identify potential Focus Care Areas for each participant. The examiner next conducted Turesky Modified Quigley-Hein Plaque Index (TMQHPI) scoring [19, 20] to quantify the extent of plaque through the whole mouth, as described below in **Clinical Evaluations**.

Following that, subjects with sufficient whole mouth plaque levels (a minimum mean of 1.75 via the TMQHPI) and one to four Focus Care Areas were then enrolled into the trial. They were provided with regular dentifrice (Blend-a-Med® Classic [1450 ppm NaF] Procter & Gamble, Gross Gerau, Germany) and asked to brush as they usually do under supervision, using their own manual toothbrush. Brushing supervisory personnel discreetly recorded the total brushing session time for each subject. Subjects were then dismissed after being directed to continue using their customary, at-home oral hygiene products and routine until their next visit.

Approximately 1 week after the screening, subjects were recalled for the baseline visit. In the same manner as at the screening visit, a comprehensive oral examination was conducted and subjects’ disclosed plaque was graded via the TMQHPI examination. All participants were then stratified according to age, gender, baseline TMQHPI scores, and number of Focus Care Areas. Subjects were randomized to one of the two (2) treatment groups using a balance and assignment software on site.

#### Interactive power brush group

Oral-B® Professional Care 6000 (D36) rechargeable power brush with Oral-B® Precision Clean brush head (EB20); charger, smartphone (Samsung® Galaxy S3; Samsung Electronics Co., Ltd., Suwon, South Korea) equipped with Oral-B® Application v2.1, OB2 phone app [Procter & Gamble, Cincinnati, OH, United States] but with restricted functionality (e.g., no SIM card).

#### Manual brush group

Oral-B® Indicator 35 soft manual toothbrush [Procter & Gamble, Cincinnati, OH, United States].

Both groups were provided with Blend-a-Med Classic (1450 ppm NaF) dentifrice to use with their assigned toothbrush; the dentifrice was overtubed to conceal product identity.

To ensure examiner blinding with respect to treatment group assignments, subjects received product usage instructions in a separate area, which specified brushing their teeth in the entire dentition for 2 min in their customary manner (manual brush group) or according to the manufacturer’s instructions, including the use of the Oral-B app (interactive power brush group). Both groups were instructed to then brush in each Focus Care Area identified for an additional 10 s. These designated areas were indicated by the Oral-B app with subject-specific settings (interactive power brush group) or via verbal instruction for the manual brush group, mimicking typical provider-to-patient practices. Subjects then brushed accordingly under supervision with their assigned products in front of a mirror. They were given both verbal and written instructions to continue using their assigned test products in the same manner at home twice daily (morning and evening) for the duration of the study. No other oral hygiene products were to be used during the study.

At 2 weeks post-baseline, subjects returned for the final visit, where the comprehensive oral examination and disclosed plaque TMQHPI evaluations were performed as previously by the same examiner who was blind to treatment. Subjects were directed to brush under supervision in the same manner as they had done at home in the preceding two-week treatment phase with their assigned toothbrush and regular paste, while clinic staff discreetly recorded the duration of toothbrushing time.

The primary outcome measure was the change from baseline at Week 2 in the group mean plaque score, as evaluated by TMQHPI measurements. A secondary outcome measure was the change from baseline at Week 2 in the group mean plaque scores in specified Focus Care Areas, as evaluated by TMQHPI measurements. An additional secondary outcome measure was change in mean toothbrushing duration (measured in seconds) from baseline at Week 2.

### Clinical measurements

#### Individual focus care areas

**Fig. 1:**
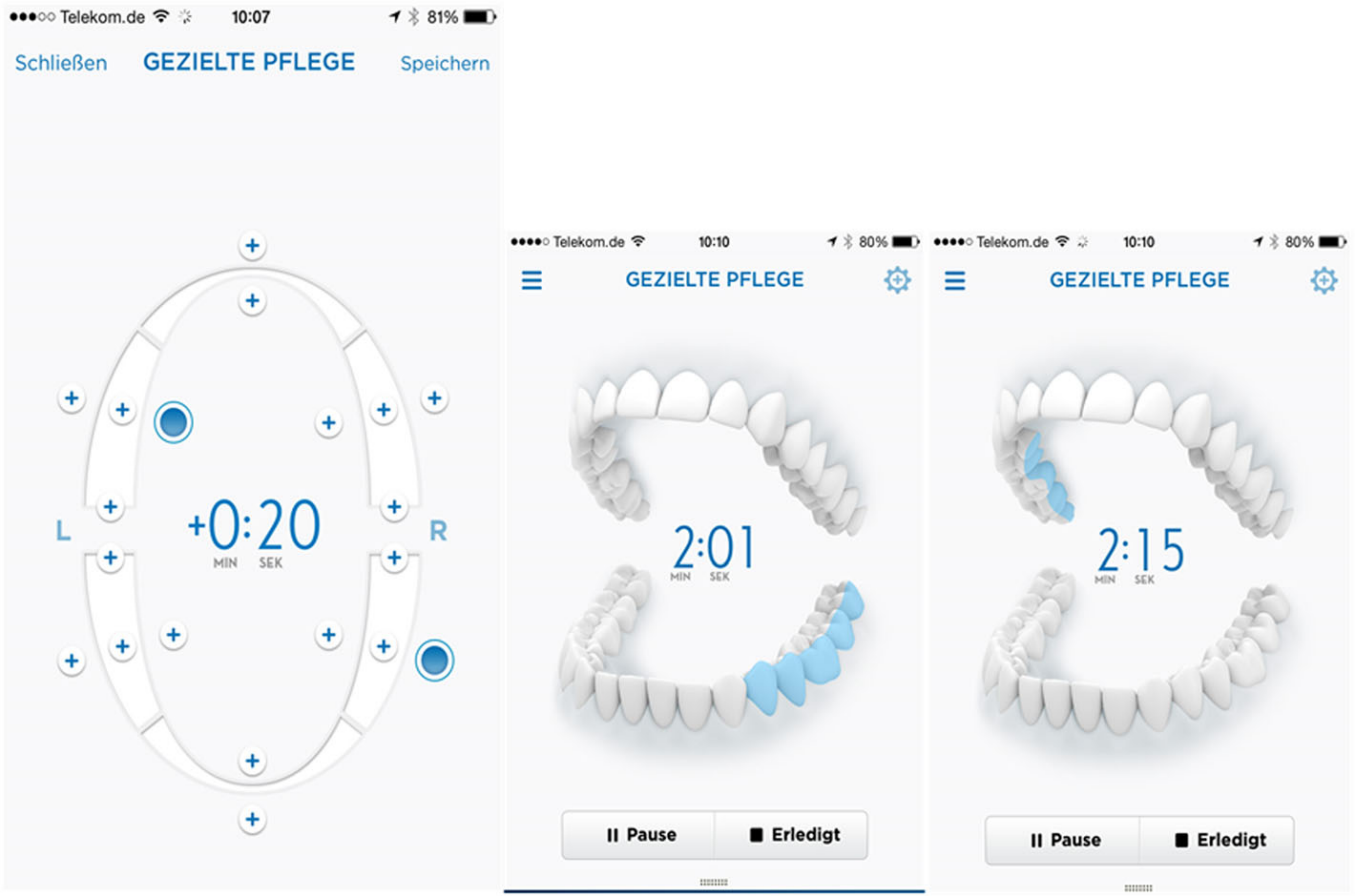
Example of display of the Oral-B Smartphone application in the “Settings Mode” (A) for “Focus Care” areas and in “Brushing Mode” (B and C)

The subjects’ dentition was classified in sextants into front teeth (incisors and canines, with subareas upper lingual, lower lingual, upper facial, and lower facial) and back teeth (premolars and molars, with subareas upper right lingual, upper right buccal, upper left lingual, upper left buccal, lower right lingual, lower right buccal, lower left lingual, and lower left buccal). Individual Focus Care Areas were designated where, in the clinical examiner’s judgment, there was a considerable amount of plaque present, indicating the need for improvement in oral hygiene. Subjects in both groups were directed to brush each of their identified Focus Care area teeth for an additional 10 s after whole mouth brushing at each treatment phase brushing session. For those assigned to the interactive power brush group, the app functioned like an interactive reminder prompting subjects via pictograms to brush additional requested time in each Focus Care Area after reaching the required two-minute whole mouth brushing (see Fig. 1).

#### Turesky modified Quigley-Hein plaque index

Plaque evaluations were performed by an experienced examiner using the Turesky modification of the Quigley-Hein Plaque Index (TMQHPI) [19, 20]. With this method, the disclosed plaque was scored using a 0–5 scale on two sites per tooth (facial and lingual) of all natural teeth except third molars, and on up to 56 potential dental surfaces. A mean plaque score was then derived for each subject at each examination by summing the individual plaque scores (two per tooth) and dividing that sum by the number of sites graded for that subject. From the addition of all individual scores, average group plaque scores and standard errors were calculated. Plaque formation was scored using the following criteria:

‘0’ = No plaque present.

‘1’ = Separate flecks of plaque at the cervical margin.

‘2’ = A thin continuous back of plaque (up to 1 mm) at the cervical margin.

‘3’ = A band of plaque wider than 1 mm but covering less than one-third of the side of the crown of the tooth.

‘4’ = Plaque covering at least one-third but less than two-thirds of the side of the crown of the tooth.

‘5’ = Plaque covering two-thirds or more of the side of the crown of the tooth.

### Statistical analyses

A sample size of 30 subjects per group in this study yielded more than 90% power to detect a treatment difference in whole mouth plaque reduction of 0.266 with the observed variability of 0.312, using two-sided testing at the 5% significance level. Summary statistics of the demographic characteristics and baseline variables were summarized by treatment group. Statistical analyses for plaque efficacy were based on average whole-mouth TMQHPI change from baseline score. The Week 2 plaque reduction was analyzed for treatment differences using an analysis of covariance method (ANCOVA) with baseline whole mouth TMQHPI score as the covariate using SAS version 9.4. Similar analyses were carried out for determining treatment differences for plaque reduction in the identified Focus Care Areas. The within-treatment group difference from baseline scores for each endpoint was tested versus zero using a paired t-test.

The brushing times (in seconds) collected at the Screening and Week 2 visits were summarized, and the change from the screening visit times were analyzed for between-group differences using the Wilcoxon’s Rank Sum test. All statistical comparisons conducted were two-sided at the 0.05 level of significance.

## Results

### Subject demographics

**Fig. 2:**
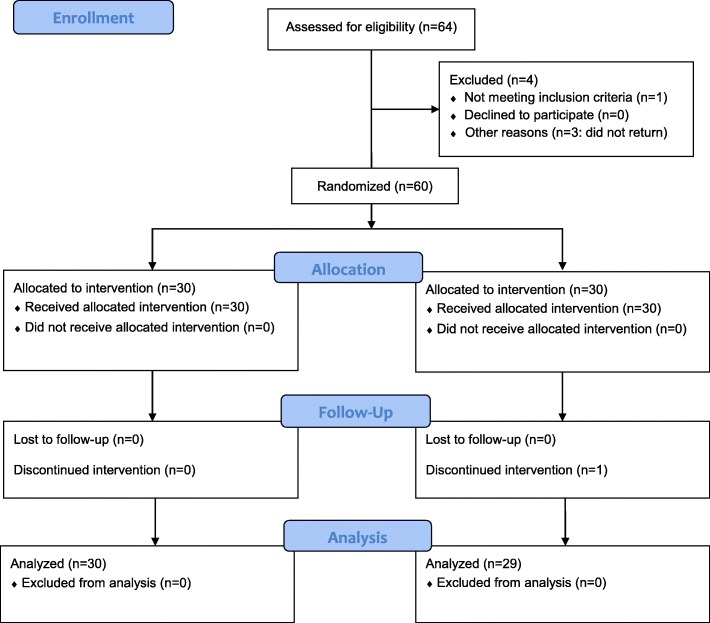
Subject flow diagram

A total of 64 subjects were screened and 60 subjects were randomized at the baseline visit, with 30 subjects assigned to the interactive power brush group and 30 to the manual toothbrush control group (Fig. 2).

One subject in the interactive power brush group was withdrawn for lack of compliance, leaving 59 subjects (98%) who completed the trial and had fully evaluable data. The mean age of the randomized study population was 15.3 years, with a range of 13 to 17 years. Sixty-three percent (63%) of participants were female, and 90% were Caucasian. The groups were not significantly different with respect to any of the baseline demographic variables (*p* ≥ 0.237; Table 1).

**Table 1:** Baseline Subject Characteristics (Randomized Subjects)

	Interactive power brush	Control	Total
Characteristic	*N* = 30	*N* = 30	*N* = 60
Mean Age^a^ (SD)	15.3 (1.02)	15.3 (1.21)	15.3 (1.11)
Age Range	14–17	13–17	13–17
Female (N, %)^b^	19 (63.3%)	19 (63.3%)	38 (63.3%)
Male (N, %)^b^	11 (36.7%)	11 (36.7%)	22 (36.7%)
Caucasian (N, %)^c^	28 (93.3%)	26 (86.7%)	54 (90.0%)
Non-Caucasian (N, %)^c^	2 (6.7%)	4 (13.3%)	6 (10.0%)

N = number of subjects; % = percentage of subjects

^a^Two-sample t-test was used to compare mean age between the two groups (*p* = 1.000).

^b^Chi-Square test was used to assess balance between the two groups for gender (*p* = 1.000).

^c^Fisher’s Exact test was used to assess balance between the two groups for race (*p* = 0.237)

### Baseline data

Prior to treatment, the baseline pre-brushing whole mouth mean TMQHPI plaque scores did not differ significantly between groups (*p* = 0.765), with a mean of 2.554 for the interactive power brush group and 2.532 for the manual brush control group (Table 2). Similarly, when analyzed in the Focus Care areas, the mean TMQHPI pre-treatment plaque scores of 3.127 for the interactive power brush group and 3.211 for the manual brush group, respectively, were not significantly different (*p* = 0.363).

**Table 2:** Pre-Treatment Mean TMQHPI Results for Evaluable Subjects

	Mean	Standard deviation	*p*-value^a,c^
Whole Mouth			
Interactive Power Brush (*n* = 29)	2.554	0.3160	0.765^a^
Manual Brush (*n* = 30)	2.532	0.2375	
Focus Care Areas^b^ Only			
Interactive Power Brush (*n* = 29)	3.127	0.3878	0.363^c^
Manual Brush (*n* = 30)	3.211	0.3150	

*TMQHPI* Turesky Modification of the Quigley-Hein Plaque Index

^a^Test group comparison at baseline using a two-sided t-test

^b^Each subject had 1–4 Focus Care Areas identified at the screening visit

^c^Test group comparison at screening using a two-sided t-test

**Fig. 3:**
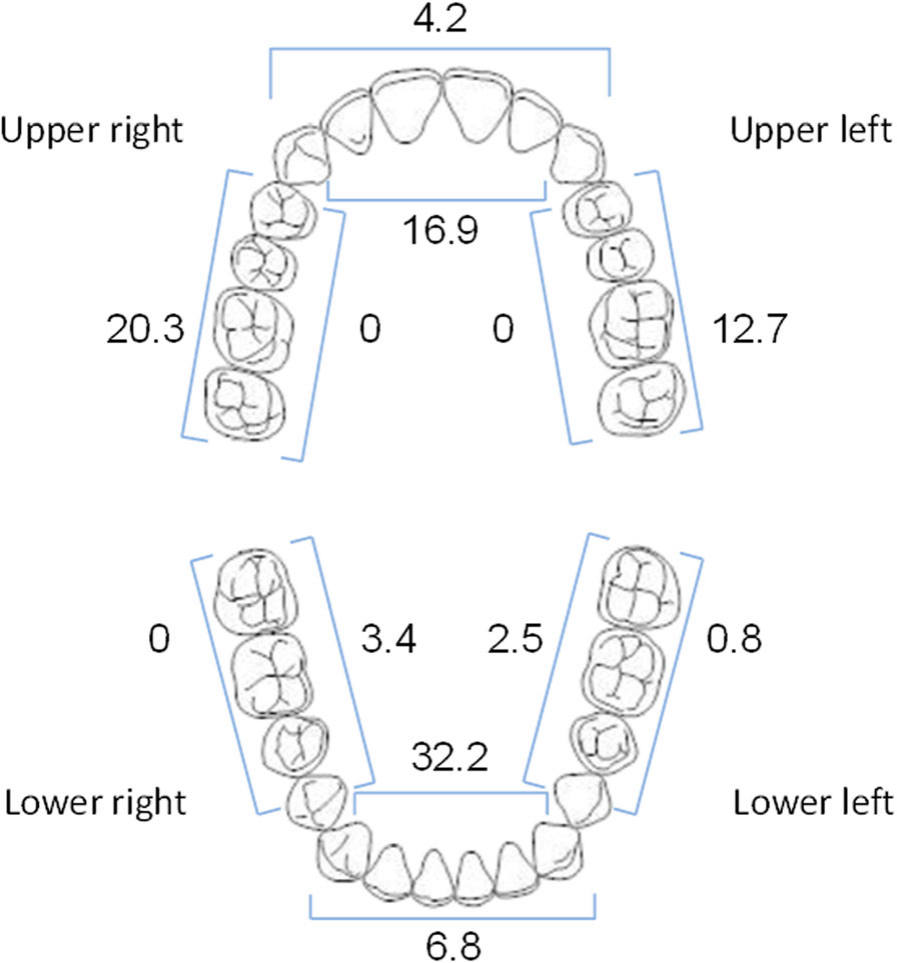
The distribution of Focus Care Areas (FCA) in the subject population by region. Numbers represent the frequency (percentage) of FCAs in each area

With respect to Focus Care Areas, prior to treatment there were 118 Focus Care Areas identified in the 59 evaluable subjects, with 32.2% observed on the lingual surfaces of the lower front teeth, 20.3% on the buccal surfaces of the upper right molar teeth, 16.9% on the lingual surfaces of the upper front teeth, and 12.7% on the buccal surfaces of the upper left molar teeth (Fig. 3). In all other regions, the frequency of observed Focus Care Areas was ≤ 6.8%.

A similar distribution was observed when analyzing the percentage of subjects who had a Focus Care Area in a certain region of the mouth: 64.4% had a Focus Care Area on the lingual surfaces of the lower front teeth, followed by 40.7% on the buccal surfaces of the upper right molar teeth, 33.9% on the lingual surfaces of the upper front teeth, 25.4% on the buccal surfaces of the upper left molar teeth, and 13.6% on the facial surfaces of the lower front teeth. In all other regions, the frequency of subjects with Focus Care Areas was ≤ 8.5%.

### Efficacy results

As shown in Table 3, following two weeks of brushing with the assigned toothbrush, subjects assigned to the interactive power brush group experienced a statistically significant 34% overall plaque reduction from baseline (*p* < 0.001), with an adjusted mean whole mouth TMQHPI change from baseline of 0.865. Subjects using the manual brush had an adjusted mean whole mouth plaque reduction of 0.044 (1.7% reduction) from baseline, which was not statistically significant (*p* = 0.231). The between-group difference in whole mouth plaque reduction favoring the interactive power brush group was highly statistically significant (*p* < 0.0001).

**Table 3:** Week 2 Whole Mouth Mean TMQHPI Efficacy Results for Evaluable Subjects

	Adjusted mean change from baseline (SE)	% Change from baseline^a^	*P*-value^b^
Interactive Power Brush (*n* = 29)	0.865 (0.0587)	34.0%	< 0.001
Manual Brush (*n* = 30)	0.044 (0.0363)	1.7%	0.231

*TMQHPI* Turesky Modification of the Quigley-Hein Plaque Index; *SE* standard error; % = percentage

^a^% change from baseline = 100% (Adjusted mean change divided by overall baseline mean)

^b^Within brush difference from baseline TMQHPI was tested versus zero using the ANCOVA model with unequal variances

When analyzed for plaque in Focus Care Areas only, both of the groups experienced a statistically significant improvement in mean TMQHPI scores compared to baseline (*p* ≤ 0.001), with an average plaque reduction of 1.208 and 0.197 for the interactive power brush and manual brush groups, respectively. The magnitude of the plaque reduction in the Focus Care Areas, however, was markedly and statistically significantly greater in the interactive power brush group (38.1%) relative to baseline than the 6.2% mean improvement in the control group (Table 4).

**Table 4:** Week 2 Focus Care Areas Mean TMQHPI Efficacy Results for Evaluable Subjects

	Adjusted mean change from screening (SE)	% Change from screening^a^	*P*-value^b^
Interactive Power Brush (*n* = 29)	1.208 (0.0893)	38.1%	< 0.001
Manual Brush (*n* = 30)	0.197 (0.0526)	6.2%	0.001

*TMQHPI* Turesky Modification of the Quigley-Hein Plaque Index, *SE* standard error, *%* percentage

^a^% change from screening (pre-treatment) = 100% (Adjusted mean change divided by overall Focus Care Areas screening mean)

^b^Within brush difference from screening TMQHPI was tested versus zero using the ANCOVA model with unequal variances

Analyses of the recorded brushing times during supervised onsite sessions showed the screening visit average brushing time was 108.6 s - with a median of 94 s - for evaluable subjects who were later assigned to the interactive power brush group, and 119.2 s - with a median of 118 s - for subjects who were ultimately assigned to the manual brush control group. Subjects were not stratified on baseline brushing time, and while the difference between groups was not statistically signficant, it was directionally different with the manual group having a longer baseline mean brushing time (*p* = 0.057; Table 5). Following two weeks of brushing with the interactive power brush, the mean brushing time for that group increased to 143.5 s (*p* < 0.001 when compared to baseline), with a median 140 s. In contrast, the mean brushing time for the manual brush control group remaining essentially unchanged after two weeks of brushing, with a mean of 118.4 s (*p* = 1.00 when compared to baseline) and a median of 111 s. When the two groups were directly compared, the change in brushing time, as reflected by increased seconds of brushing at Week 2 for the interactive power brush group relative to the control group, was statistically significant (*p* < 0.001; Table 5).

**Table 5:** Mean Brushing Times Results for Evaluable Subjects

	Mean in seconds^a^	Median in seconds	*p*-value^b,c^
Screening (Pre-Treatment)			
Interactive Power Brush (*n* = 29)	108.6	94	0.057^b^
Manual Brush (*n* = 30)	119.2	118	
Week 2			
Interactive Power Brush (*n* = 29)	143.5	140	< 0.001^c^
Manual Brush (*n* = 30)	118.4	111	

^a^Subjects’ brushing time in seconds was recorded during a supervised brushing session

^b^Between-group comparison of screening brushing times for subjects ultimately assigned to the test groups, using a Wilcoxon’s Rank Sum Test

^c^Between-group comparison of the change in brushing time (Week 2 minus screening) using a Wilcoxon’s Rank Sum Test

### Safety Results

There were no adverse events reported or observed over the course of the clinical trial.

## Discussion

As mobile phone ownership and app use continue to show exponential growth, so does the popularity of health apps for lifestyle and medical monitoring. A projected half billion people will use healthcare mobile apps in 2015, and by 2018 this will swell to 50% of the estimated 3.4 billion tablet and smartphone users [21]. Many health-promoting apps are carefully designed to give a user-friendly, engaging experience to encourage regular usage. Outcomes-based research across a diverse array of health conditions and disciplines, such as diabetes, asthma, dementia, smoking cessation, and weight loss intervention, suggests that app usage may indeed produce measurable, cost effective results [22]. A variety of delivery modes have been employed to communicate health information, including text reminder messages, self-report questionnaires, physical activity tracking, and phone camera data transmission. Well-designed health apps can be a highly effective adjunct to health care delivery by facilitating self-motivation, targeted behavior modification, and/or professional real time monitoring. In essence, mobile devices can serve as portable, advanced computers for remotely tracking and accessing health information for wellness and disease management, while the apps simultaneously deliver the individualization that wireless technology users have come to value. In the realm of oral health and dental disease, mobile phone apps have been less proliferative but availability is increasing. Several free or low-cost apps educate patients on dentistry and dental procedures (e.g., #1 Dental Expert, ADA Symptom Checker) or teach general oral hygiene (Cavity Free 3D, Dental Care Aid). Others target motivation: the Brush DJ app for example, which plays user playlist music for two minutes to maximize brushing time, was self-reported to be efficacious in the majority of volunteers in one recent investigation [23].

One of the more advanced functionalities in mobile phone app technology, which is used in the clinical trial reported here, is that of Bluetooth, a wireless technology facilitating data exchange between mobile and other electronic devices. Combined with the advanced Oral-B power brush, this innovative interactive capability in oral health applications allows users to have highly personalized instant feedback specific to their oral hygiene needs, all within a fun-to-use experience and via a device they consider indispensable. This technology also provides an opportunity for the dental professional and patient to jointly review actual brushing data, discuss oral hygiene progress and opportunities to optimize the brushing experience, and set appointment reminders. An adolescent population was targeted for this two-week, randomized and controlled clinical trial, given teens’ known high utilization of mobile technology, as well as their greater risk for dental disease during this life stage. If adolescents generally care about their grooming, it may seem counterintuitive that an unacceptably high percentage invest little time and effort into daily oral hygiene. However, several potentially contributing factors are at play in the adolescent years. Teens may have insufficient awareness of the direct connection between thorough plaque removal and gingivitis and caries prevention, or fail to appreciate that neglecting good hygiene for their now-permanent teeth carries not just cosmetic, but life-long health implications. Parents likely no longer routinely monitor brushing, lessening accountability, while teens may not prioritize sufficient time to devote to oral hygiene, or find it boring relative to other competing interests. Regardless, in general adolescents do allocate time for mobile technology use, and recognizing this, this study was designed to provide insight on the benefit of combining a mobile application with a previously well-proven plaque-removing device in a population at risk for oral disease, and known for high utilization of wireless technology.

Several study design elements in this trial were designed to minimize the risk of confounding factors. With respect to subject selection, all participants were required to be former manual brush users to avert any disparity in familiarity with power toothbrush usage. Volunteers needed to be well-versed in smartphone usage to prevent usage variations resulting from any novelty factor associated with the phone and app. To avoid bias in data collection, subject brushing times were recorded by clinical site personnel without the participants’ awareness, for a better representation of actual home usage. The test products also reflected “real-world” product availability. Use of Bluetooth technology with toothbrushes is currently limited to a few power toothbrush models; no manual toothbrushes have this capability yet. Coupled with the fact that manual toothbrushes are the most widely used oral hygiene device, the choice of a manual control without Bluetooth technology was appropriate.

The Oral-B family of oscillating-rotating power toothbrushes has now been evaluated for efficacy in more than 100 in vitro and over 150 clinical trials, with published results establishing benefits in removing plaque and improving gingival health [24]. Large independent meta-analyses show statistically significant greater plaque- and-gingivitis-fighting efficacy compared to manual brushes in short and long-term clinical studies, and relative to other power brushes with side-to-side action [25, 26]. The results reported here are consistent with previous research, confirming the superiority of the Oral-B Professional Series oscillating-rotating brush - maximized with the app - against a manual toothbrush for plaque reduction in this population of German adolescents. Only the interactive power brush delivered a statistically significant whole-mouth plaque reduction after 2 weeks of brushing, and the magnitude of the benefit was substantial, with a 34% mean decrease from baseline. Both brushes provided statistically significant mean plaque reduction in the Focus Care Areas, but the magnitude of the benefit in these regions was markedly and statistically significantly greater in the interactive power brush group (38.1%) relative to baseline than the 6.2% mean improvement in the manual brush control group (*p* < 0.001).

Patient compliance with dental professional recommendations around optimal home oral hygiene is notoriously inconsistent. By adolescence, most have heard repeatedly that they should brush their teeth for at least 2 min with a fluoride toothpaste, yet research shows few comply [2–4]. Collectively these results demonstrate striking oral health benefits when patients are motivated by advanced oral hygiene products and technologies to brush longer and more thoroughly– in this case via an interactive power toothbrush – as compared to a typical manual brushing routine.

One limitation in the current study is that the subject population as a whole averaged 113.9 s brushing time at study inception, much longer than the typical average brushing time of 1 min or less [2–4]. This may be due to the Hawthorne effect, which purports that subjects’ behavior is modified as a result of knowing they are participating in an investigation [27]. Despite this, the results after 2 weeks of brushing revealed that subjects in the Oral-B interactive power brush group demonstrated an increase of 34.9 mean brushing time seconds per session, a statistically significant 32% increase from baseline. In contrast, subjects assigned to the manual brush group showed essentially no change in mean brushing time (a non-significant 1% decrease from baseline). Several aspects may have contributed to the longer brushing time with the power toothbrush, including interactivity, personalization, and the Focus Care area reminders. Subjects in the manual brush group, conversely, were dependent on their recall of the instructions given to them previously about their particular Focus Care Areas and the need for extra follow-up brushing in those locations, as is the case in actual typical provider-patient 6-month recall visit situations. An interesting topic for future research would be to extend the duration of the study to 6 months or longer and assess efficacy and compliance outcomes.

Dental plaque formation has been shown to be predictably heaviest in more difficult to access areas [28]. Any regions that are challenging in requiring more skill to reach and/or time and attention with the toothbrush are susceptible to heavier plaque and calculus formation, and at greater risk for caries and gingivitis when neglected [29, 30]. As displayed in Fig. 3, the overall distribution of clinician-identified Focus Care Areas in this trial showed the highest frequency in the mandibular anterior lingual regions, followed by the upper right buccal surfaces. The remaining Focus Care Areas were distributed in smaller percentages in multiple regions across the dentition, likely owing to differences in right- or left-handedness and individual variations in dexterity. This highlights the importance of a customized, personal oral hygiene action plan to encourage extra brushing concentration in areas most at risk for disease. As shown by the study results, a technology-based reminder via a platform that is already used regularly can provide an excellent means of supplying these ongoing, daily prompts, with the result that brushing times are lengthened, and plaque reduction is heightened.

## Conclusions

In this two-week, randomized, single-blind study, adolescents using an interactive power toothbrush with Bluetooth technology exhibited statistically significantly greater plaque reduction both overall and in specified Focus Care Areas, as well as a longer duration of brushing time, than did adolescents using a manual toothbrush. An interactive power toothbrush with Bluetooth technology appears to appeal to technology-savvy adolescents, producing increases in brushing efficacy, duration, and compliance among this vulnerable population.

## Abbreviations

FCA: Focus Care Areas
IPT: Interactive Power Toothbrush
MT: Manual Toothbrush
NHANES: National Health and Nutrition Examination Survey
TMQHPI: Turesky Modified Quigley-Hein Plaque Index
